# A Comprehensive Review of Medial Thighplasty: The Role of Liposuction in Reducing Complications and Optimizing Patient Outcomes

**DOI:** 10.3390/jcm14072426

**Published:** 2025-04-02

**Authors:** Roberta Albanese, Claudio Gio Francesco Blessent, Federica Tomaselli, Giorgio De Santis, Valentina Pinto, Massimo Pinelli, Ernesto Maria Buccheri, Damiano Tambasco

**Affiliations:** 1 Plastic Surgery Unit, San Carlo of Nancy Hospital, GVM Care and Research Group, 00185 Rome, Italydamianotambasco@gmail.com (D.T.); 2 Department of Plastic and Reconstructive Surgery, Ospedale Santa Maria della Misericordia, 33100 Udine, Italy; 3 Department of Plastic Surgery, Policlinico di Modena, University of Modena and Reggio Emilia, 41124 Modena, Italy; claudio.blessent93@gmail.com (C.G.F.B.);; 4 Private Practice, Medicinaplasticaroma Center, Plastic Surgery, Via Clitunno 22, 00198 Rome, Italy

**Keywords:** medial thighplasty, liposuction, complications

## Abstract

**Background/Objectives:** Medial thighplasty is a widely performed body contouring procedure, particularly in patients with massive weight loss. Despite advancements in surgical techniques, complications remain a significant concern. The integration of liposuction into thigh lift procedures has shown promise in improving both aesthetic and functional outcomes while reducing risks. This review aims to assess the role of liposuction in medial thighplasty by evaluating its impact on surgical outcomes and complications. **Methods**: This systematic review was conducted following PRISMA guidelines. A comprehensive search was performed in the MEDLINE/PubMed database using predefined keywords. A total of 52 records were identified, of which 19 studies met the inclusion criteria, encompassing 1113 patients who underwent liposuction-assisted medial thighplasty. Two independent reviewers screened and selected the studies, resolving discrepancies through discussion or consultation with a third reviewer. Data on surgical techniques, complications, and outcomes were extracted and analyzed. Statistical comparisons were performed using the Chi-square test, with significance set at *p* < 0.05. **Results:** The incorporation of liposuction significantly reduced the overall complication rate (36.75% vs. 70.68%, *p* < 0.001). Specific complications, including infections (1.77% vs. 9.02%), hematomas (1.30% vs. 6.77%), and seromas (8.95% vs. 24.81%), were markedly lower in the liposuction group. **Conclusions**: Liposuction appears to reduce complication rates and improve surgical outcomes in medial thighplasty. Further standardization of techniques and additional research on advanced liposuction technologies are necessary to refine this surgical approach and optimize patient outcomes.

## 1. Introduction

In recent years, there has been growing interest in body contouring, driving the development of medial thighplasty techniques specifically tailored to the diverse needs of different patient populations [1,2,3].

An innovative approach tailors the intervention to the patient’s clinical conditions, expectations, and surgical feasibility. Patients with massive weight loss (MWL) require specific strategies due to tissue damage from weight fluctuations and significant excess tissue [4,5].

Currently, medial thighplasty techniques often utilize a combination of liposuction, skin excision, and skin thightening technologies [6,7].

In literature, different surgical technique are described: horizontal [8,9,10,11,12,13,14,15,16], vertical [10], T-shaped [8,10,11,12,13,15,17,18,19,20,21], and L-shaped [22,23,24,25], allowing for individualized solutions based on each patient’s needs. The goal remains to achieve an aesthetically pleasing thigh contour with minimal scarring and a low risk of complications [26].

The initial advancements in thigh lift surgery focused on fascial anchoring techniques, providing enhanced stability and contouring [16]. In recent years, less invasive approaches have been favored, utilizing liposuction to avoid breaching deep planes, thereby minimizing invasiveness [27].

The introduction of liposuction as an adjunct has marked a significant milestone, allowing for improved contouring, reduced tension, and minimized surgical trauma. This progression reflects a commitment to optimizing patient safety and aesthetic results while addressing the complexities of thigh lift procedures.

Its introduction, in conjunction with the horizontal technique, has made it possible to avoid posterior incisions in the gluteal fold and extensions into the gluteal crease, while maintaining a uniform height [9,28]. Instead of weakening the area, they performed comprehensive liposuction across the entire thigh, particularly in regions planned for skin resection. Their approach preserved lymphatic and vascular structures by removing only the skin and not the underlying liposuctioned tissue, thereby reducing complications [9].

Over the following 20 years, thighplasty techniques increasingly incorporated liposuction due to its undeniable advantages. However, comparative studies highlighting its specific benefits are still lacking.

This study aims to conduct a comprehensive review of the literature on medial thighplasty, focusing specifically on the role of liposuction and its potential advantages and benefits in the execution of this surgical procedure. By examining the available evidence, the study seeks to determine whether the integration of liposuction enhances aesthetic and functional outcomes, minimizes complications, or optimizes tissue contouring.

## 2. Materials and Methods

This systematic review was conducted in accordance with the Preferred Reporting Items for Systematic Reviews and Meta-Analyses (PRISMA) guidelines. The objective was to evaluate the role of liposuction in medial thighplasty by systematically reviewing the available literature.

### 2.1. Search Strategy

A comprehensive literature search was conducted in the MEDLINE/PubMed database in September 2024. The following Boolean search strategy was applied: ((“lipothighplasty” [All Fields]) OR (“thigh lift procedure” [All Fields]) OR (“thighplasty” [All Fields]) OR (“thigh lift” [All Fields])) AND ((“liposuction” [All Fields]) OR (“lipoaspirate” [All Fields])).

Additionally, the reference lists of selected articles were screened to identify additional relevant studies.

### 2.2. Eligibility Criteria

The inclusion and exclusion criteria were defined as follows:

- Inclusion Criteria:
  o Original research articles on medial thighplasty, published up to September 2024.
  o Studies including patients who underwent medial thighplasty.
  o Prospective and retrospective observational studies, including case series.
  o Studies evaluating complications and surgical outcomes.
- Exclusion Criteria:
  o Articles not written in English.
  o Case reports, editorials, expert opinions, systematic reviews, meta-analyses.
  o Studies with insufficient or incomplete data on surgical outcomes.

### 2.3. Study Selection

**Figure 1:**
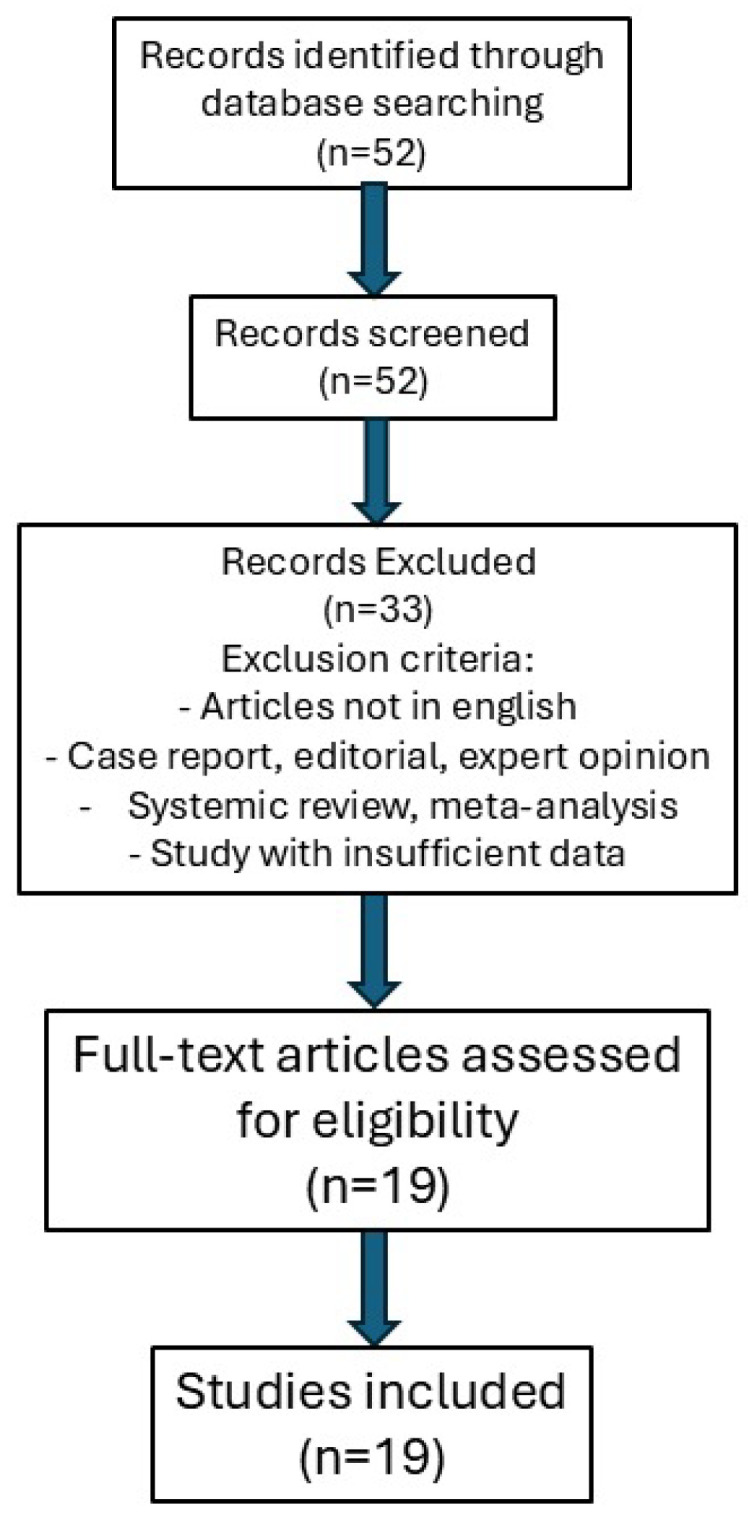
PRISMA flow diagram of the study selection process.

A total of 52 records were identified through database searching. Two independent reviewers screened the titles and abstracts of all retrieved studies. A total of 33 studies were excluded. The remaining 19 full-text articles were assessed for eligibility. Articles that met the inclusion criteria underwent full-text review, and any discrepancies between the reviewers were resolved through discussion or, if needed, by a third reviewer. No additional exclusions were made at this stage, and all 19 studies were included in the final review. The study selection process is summarized in the PRISMA flow diagram (Figure 1).

Information on authorship, publication date, patient numbers, surgical techniques, and complications were extracted from the selected articles (Table 1).

To simplify the description, we decided to classify the techniques based on the following characteristics.

Medial thighplasty techniques:

- Horizontal pattern: This was the first thigh lift technique described by Lewis [28], later improved by Lockwood [16] with fascial anchoring, and further refined by Le Louarn and Pascal [9], who introduced the use of liposuction to minimize wide undermining and reduce complications. This approach involves a horizontal incision along the inguinal crease, extending laterally toward the hip. It is best suited for patients with minimal laxity in the upper thigh, allowing for removal of excess skin and improved contours in the upper inner thigh [9,10,11,12,13,14,16,17].
- Vertical pattern: This method involves a vertical excision of excess tissue from the inguinal crease to or near the knee, often accompanied by a horizontal extension into the inguinal crease to remove excess skin. It is indicated for patients with more extensive laxity, allowing for more skin removal and more complete contouring [2,4,5,9,11,12,13,14,15,16,17,18,19,20,21,22,23].

**Figure 2:**
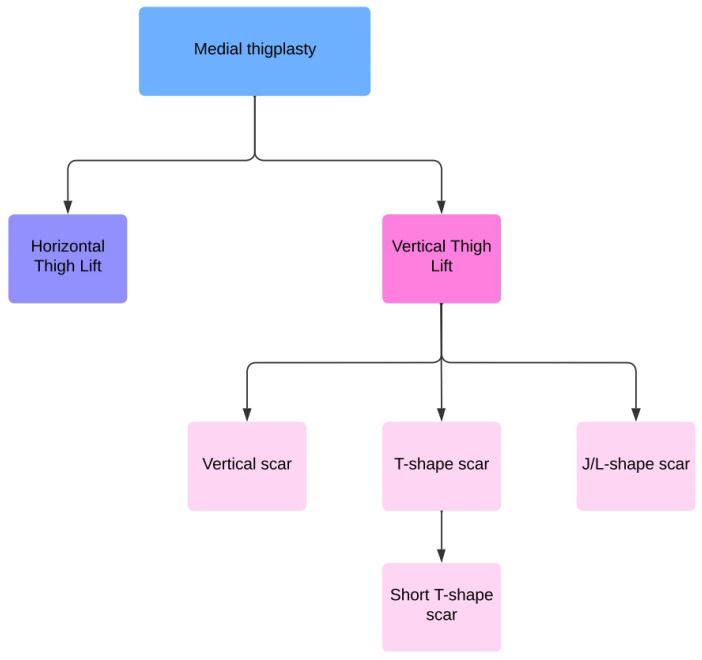
Vertical Medial Thighplasty classification based on the scar’s design.

Depending on the scar’s design, vertical techniques can be classified into (Figure 2):

- Vertical scar: This technique involves an excision that leaves a vertical scar extending from the groin to the knee along the inner thigh [10].
- T-shape scar: This is the classic approach that combines a vertical incision with a horizontal incision in the inguinal fold, forming a “T”. It addresses both vertical and horizontal laxity.
- L/J-shape scar: This technique addresses extensive laxity with the “L” or “J” shape conforming to thigh anatomy and reducing tissue tension. It shows a lower complication rate compared to the T-technique [22,23,24,25].
- Short T-shape scar: This variation aims to minimize the horizontal scar length, potentially reducing complications seen with the traditional T-technique [12].

The articles were further categorized into two distinct groups to better analyze the differences between approaches. Group I included standard techniques that did not involve the use of liposuction, focusing solely on the removal of excess tissue through traditional methods. In contrast, Group II comprised techniques that integrated liposuction, allowing for a more comprehensive contouring by addressing both excess skin and underlying fat. This differentiation was essential to evaluate the advantages and limitations of each approach in achieving optimal outcomes.

## 3. Results

We identified 52 full-text articles; 33 did not meet inclusion criteria, leaving 19 studies available.

In total, 1113 patients were included across the 19 studies analyzed.

The distribution of techniques was as follows: 153 horizontal medial thigh lifts, 15 vertical thigh lifts, 757 vertical T-shaped thigh lifts, and 188 vertical L/J-shaped thigh lifts.

The follow-up period across studies ranged from 15 days to 9.5 years.

For the horizontal technique, all procedures incorporated fixation to the Colles’ fascia or periosteum. In contrast, the vertical technique showed variability among authors: some did not perform medial fixation, citing the presence of a horizontal tension vector as described by Mathes [8], while others opted for fixation to the periosteum or Colles’ fascia for enhanced stability.

**Figure 3:**
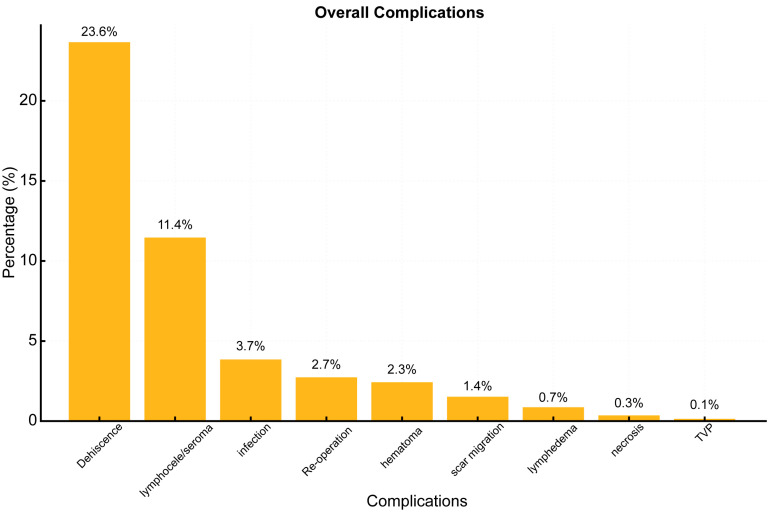
Overall complication.

Pooling the data, complications were observed in 483/1113 patients (46.3%). The analysis of total complication (Figure 3) rates highlights that dehiscence is the most frequent complication, affecting 23.6% of all patients. This is followed by lymphocele/seroma (they have been grouped into the same category because they are clinically indistinguishable [13]) at 11.4% and infections at 3.7%. Less frequent complications include re-operation (2.7%) and hematoma (2.3%). Although necrosis has a low incidence (0.3%), a clear distinction between necrosis and dehiscence could not be established. Furthermore, there is no standardized approach to differentiating between the two conditions or their management. The Deep Vein Thrombosis (DVT) is the least frequent complication observed (0.1%). Lymphedema is an uncommon complication (0.7%). However, none of the articles utilized objective assessments with instrumental examinations, such as lower limb lymphoscintigraphy, in cases of residual edema. This suggests that the reported incidence may be underestimated.

The analysis revealed a significantly lower overall complication rate in patients who underwent liposuction compared to those who did not (36.75% vs. 70.68%, *p* < 0.001). Additionally, the incidence of infection, hematoma, scar migration, and seromas was markedly reduced in the liposuction group compared to the non-liposuction group (Table 2).

## 4. Discussion

Thigh lift is one of the most commonly performed procedures in body contouring surgery [31]. Traditional surgery without liposuction often allows comprehensive thigh contouring. However, various complications and limitations have been recognized over time [19].

Several designs have been described, each tailored to meet the specific needs of the patient and the objectives for achieving appropriate contouring [5,8,9,12,13,14,15,16,17,18,19,22,23,24,25,30,32].

These techniques, however, lack universal applicability and are often chosen based on the surgeon’s expertise and preferences.

The selected approach is influenced by several key factors, including the extent and location of skin laxity, the presence of lipodystrophy, and the patient’s surgical history, such as scars or tissue irregularities from previous procedures. Patient preferences also play a crucial role and should be carefully considered through open discussions about surgical options and expected outcomes, ensuring the chosen approach aligns with their goals and expectations.

Data analysis has demonstrated that incorporating liposuction significantly reduces complications (36.75% vs. 70.68%, *p* < 0.001). When integrated appropriately into the surgical approach, liposuction can minimize tissue trauma and potentially reduce the incidence of complications by preserving vital vascular and lymphatic structures [29,33]. Additionally, shorter surgical scars achieved with liposuction-assisted techniques promote faster healing and decrease complications associated with prolonged immobilization [7].

Schmidt et al. (2016) [19] conducted a retrospective cohort study comparing traditional excision-only medial thighplasty with a liposuction-assisted approach in massive-weight-loss patients. Their findings demonstrated a significant reduction in overall complication rates with the concomitant use of liposuction (13% vs. 59%, *p* < 0.001). Specifically, seroma formation (0% vs. 34%) and wound infections (3% vs. 28%) were markedly lower in the liposuction-assisted group. Additionally, these patients experienced a shorter hospital stay, fewer follow-up visits, and a faster time to drain removal, supporting the hypothesis that liposuction preserves microvasculature and reduces post-surgical morbidity. As a result, the authors abandoned the excision-only approach in their practice, emphasizing the importance of integrating liposuction into medial thigh contouring for improved outcomes.

Another study that directly compares the use of liposuction is Di Pietro et al. [21], which evaluates the LAMeT (Liposuction-Assisted Medial Thigh Lift) technique versus simple excision in massive-weight-loss patients. Their findings confirm that LAMeT significantly reduces postoperative complications, with a lower incidence of seroma (0% vs. 28%) and wound dehiscence (0% vs. 32%), compared to excision-only techniques. The key advantage of incorporating liposuction is the preservation of the lymphatic and vascular network, which minimizes the risk of chronic lymphedema and improves healing. Additionally, by avoiding extensive undermining, LAMeT accelerates recovery, leading to a shorter hospital stay (2 vs. 5 days), quicker drain removal (0.25 vs. 2.18 days), and fewer postoperative visits. The study also highlights superior aesthetic outcomes, particularly in the medial knee region, with greater long-term stability and minimal ptosis recurrence. Given these benefits, the authors completely abandoned the simple excision approach, further reinforcing the critical role of liposuction in modern medial thigh contouring.

Despite its benefits, the literature lacks consensus on the optimal liposuction strategy, including the best technology and ideal aspirate volume. However, its primary function remains the removal of excess fat and tissue mobilization, which facilitate effective contouring. This process reduces tension on wound closures, improving healing outcomes and lowering the incidence of complications like seroma, hematoma, and wound dehiscence.

Analyzing the data, the hematoma rates were significantly lower in the liposuction group (1.30% vs. 6.77%, *p* < 0.001), supporting the hypothesis that liposuction minimizes vascular trauma and postoperative bleeding. The use of tumescent solutions, which provide vasoconstriction and hydro dissection, likely contributes to this effect. Similarly, infection rates were reduced (1.77% vs. 9.02%, *p* < 0.001), likely due to improved tissue handling, reduced tension on closures, and shorter incisions. This finding is corroborated by studies conducted by Schmidt et al. [19] and Di Pietro et al. [21], where the integration of liposuction within the resection flap consistently resulted in lower infection rates. In contrast, another study by Gusenoff et al. [12] reported increased infection rates with liposuction. This discrepancy is probably attributable to the different application of liposuction; while Schmidt and Di Pietro performed liposuction directly within the tissue planned for resection—thereby preserving vascular and lymphatic structures and minimizing trauma—Gusenoff et al. [12] applied liposuction in areas outside the resection zone, which may have led to increased tissue injury and a higher risk of contamination. These observations underscore the importance of executing liposuction in a targeted manner, emphasizing that its benefits in reducing infection rates are most pronounced when it is confined to the resection flap.

The incidence of lymphocele/seroma was significantly lower in the liposuction group (2.84% vs. 23.51%, *p* < 0.001), indicating that combined techniques inflict considerably less damage to the lymphatic system, a particularly critical benefit in thigh lifts, where the complex anatomy increases the risk of lymphatic complications. This finding is consistent with the results of Schmidt, Di Pietro, and Hunstad. Similarly, Xie et al. [13] reported that techniques incorporating the use of liposuction, alongside horizontal vector fascial fixation, were associated with lower rates of lymphocele/seroma.

The lymphatic pathway, located parallel to the great saphenous vein, is particularly susceptible to damage during aggressive dissections in the medial thigh. The use of conservative techniques, including blunt cannulas, minimizes this risk and offers a clear benefit.

Additionally, the reported incidence of lymphedema complications is likely underestimated due to reliance on clinical evaluation alone and the absence of routine diagnostic tools. Subclinical or early-stage cases are challenging to identify and are often overlooked, further contributing to underreporting.

The result for wound dehiscence was not statistically significant, despite the technical advantages of liposuction in improving wound healing. Liposuction reduces tissue trauma, preserves vascularization, and decreases skin tension, all factors that contribute to better wound closure. However, the lack of statistical significance may be attributed to inconsistencies among authors in defining and classifying dehiscence.

Gusenoff et al. [12] reported the highest dehiscence rate (43%), likely due to broader criteria compared to other authors who did not specify their criteria. Schmidt et al. [19] found that in the group undergoing excision alone, dehiscence was observed in 28% of patients. However, in the group where the procedure was assisted by liposuction, the dehiscence rate was significantly lower at 3.3%. Similarly, Di Pietro et al. [20] showed a significantly reduced dehiscence rate following the use of liposuction. Surgical techniques also affect dehiscence rates, with higher values observed in T-techniques at the intersection of the scars, compared to L/J or horizontal techniques. Xie et al. [13] compared Colles’ fascia suspension fixation (CFSF) with horizontal vector fixation (HVF) in medial thigh lifts for patients with massive weight loss. The dehiscence rate in both the HVF and CFSF groups was 17.9% vs. 15.4%, showing no statistically significant difference. This study indicates that flap fixation is not associated with a reduction in dehiscence.

Standardizing the concept of dehiscence by employing objective criteria, such as the length of the affected region or the time required for healing, could prove more useful for future research.

However, there are other factors that obviously influence complications, which are data not reported or measured heterogeneously across different studies.

Risk factors play a crucial role in determining postoperative complication rates. Bertheuil et al. [23] analyzed 94 patients treated between 2014 and 2019 and found that advanced age and a BMI of 30 kg/m^2^ or higher were independent risk factors for complications, with odds ratios of 1.05 and 2.82, respectively. Additionally, Gusenoff et al. [12] reported that anemia and advanced age were associated with increased complication rates, and that hypertension was strongly linked to postoperative seroma formation. These findings underscore the importance of rigorous preoperative patient evaluation and risk stratification. In light of this, it is advisable that future protocols for medial thighplasty incorporate comprehensive screening measures to identify high-risk individuals. Future studies should focus on developing standardized risk assessment tools and investigating the efficacy of customized intervention strategies to improve outcomes in patients undergoing medial thighplasty.

It is also clear that the scar pattern plays a crucial role in determining the risk of complications in medial thighplasty. The choice between horizontal, vertical, T-shaped, or L-shaped incisions is not merely an aesthetic decision; it directly impacts the likelihood of developing complications. For example, Gusenoff et al. [12] evaluated 106 patients between 2003 and 2012 and found a significant correlation between the type of incision and the incidence of complications. Their results showed that patients undergoing full-length vertical T thighplasty experienced a complication rate of 74%, compared to 67% for short-scar thighplasty and only 43% for horizontal thighplasty.

Furthermore, Bracaglia et al. [25] explain that the combination of vertical and horizontal vectors creates a trifurcation point, which increases the risk of dehiscence and necrosis. To address these issues, the L-shaped incision was introduced as an alternative to the traditional T or vertical incisions. Studies by Bracaglia et al. [25] suggest that the L-shaped technique, when combined with selective liposuction, reduces complications by concentrating the resection in the antero-medial region of the thigh. This targeted approach avoids extending the incision posteriorly toward the perineum or infragluteal fold, thereby further decreasing the risk of dehiscence.

An additional challenge to address is the evaluation of the timeline for resuming normal physical activity. Only the study of Schmidt et al. (2016) [19] report that patients undergoing liposuction-assisted medial thighplasty experienced a faster recovery compared to those who had the traditional excision-only approach. Specifically, the liposuction-assisted group had a significantly shorter hospital stay (6.0 vs. 7.8 days, *p* < 0.001), fewer follow-up visits (2.0 vs. 4.4), and a faster time to drain removal (1.8 vs. 4.1 days, *p* < 0.001). These findings suggest that integrating liposuction into medial thigh contouring procedures not only reduces complications but also enhances postoperative recovery, allowing for quicker return to normal activities. Liposuction enables the creation of smaller scars, which significantly facilitates wound healing and leads to a faster recovery.

Another noteworthy aspect is the lack of clear guidelines on postoperative management in the reviewed studies, particularly regarding the use and effectiveness of compression garments. However, all authors agree on the importance of early mobilization to mitigate acute risks such as deep vein thrombosis (DVT) and pulmonary embolism. It is important to note that, despite the absence of standardized protocols, the incidence of DVT is remarkably low (0.1%), indicating that the preventive measures commonly employed are highly effective. Another noteworthy aspect is the lack of clear guidelines on postoperative management in the reviewed studies, particularly regarding the use and effectiveness of compression garments. However, all authors agree on the importance of early mobilization to mitigate acute risks such as deep vein thrombosis (DVT) and pulmonary embolism. It is important to note that, despite the absence of standardized protocols, the incidence of DVT is remarkably low (0.1%), indicating that the preventive measures commonly employed are highly effective.

Finally, although the specific type of technology used in liposuction is not explicitly detailed in the reviewed studies, it is evident that advanced techniques capable of minimizing bleeding and preserving the fibroseptal network could offer significant advantages. These benefits include improved wound healing and enhanced thigh contouring, particularly with comprehensive 360° techniques.

Moreover, while not yet described in the literature, the incorporation of additional technologies aimed at improving skin tightening could address some limitations of the procedure, such as scar migration, which remains a challenge in current practice. These advancements could further refine outcomes and optimize patient satisfaction.

## 5. Conclusions

Thigh lifting is a cornerstone of body contouring, particularly for patients with significant weight loss or severe skin laxity. This analysis underscores the importance of addressing complications, refining surgical techniques, and tailoring approaches to individual patient needs.

The significant reduction in overall complications observed with liposuction highlights its transformative role in thigh lift procedures. Beyond contouring, liposuction reduces tissue trauma, preserves vascular and lymphatic integrity, and enhances wound healing. This dual aesthetic and functional benefit is further supported by lower rates of specific complications, such as hematoma, infection, and lymphocele/seroma.

## 6. Limitations

One of the major limitations of this review is the heterogeneity of the included data. The analyzed studies presented significant variations in several key factors, including patient selection, surgical techniques, and follow-up periods (ranging from 15 days to 9.5 years). Furthermore, the reviewed literature did not provide consistent definitions for wound dehiscence, nor did it differentiate between the various liposuction techniques (wet, super-wet, suction-only, water-assisted, vibration-assisted, laser-assisted29), which may have a significant impact on the treated tissue and, consequently, on complication rates. Additionally, the application of liposuction varied, with some studies performing it over the entire thigh and others only in the resected area, further contributing to data heterogeneity.

Another relevant limitation is the comparison of complication rates across a heterogeneous group of patients undergoing different surgical techniques (e.g., horizontal lifting, vertical lifting, J-lifting). Since these procedures have varying degrees of invasiveness, direct comparisons may not fully account for differences in surgical complexity and tissue handling.

Given these inconsistencies, conclusions should be interpreted with caution. Future research should prioritize a standardized methodological approach to enhance comparability while also refining surgical techniques and exploring the nuances of liposuction, including technology types, aspirated volumes, and their effects on outcomes. Larger, well-designed studies are crucial to validate these findings and refine best practices. Incorporating advanced liposuction technologies, such as ultrasound or power-assisted systems, may further enhance precision and safety, ensuring thigh lift procedures remain effective and reliable for addressing thigh laxity.

Another important limitation of this review is the lack of systematic consideration of patient comorbidities. Comorbid conditions, such as obesity, diabetes, hypertension, smoking habits, and cardiovascular disease, can significantly influence surgical outcomes and complication rates in medial thighplasty procedures. Since most studies reviewed did not systematically evaluate or report these factors, it was impossible to stratify the results accordingly. Future research should prioritize detailed reporting of comorbidities, and ideally, perform subgroup analyses based on these patient-specific risk factors, in order to provide clearer guidance for clinical practice.

By integrating these insights with individualized care, surgeons can continue to improve outcomes, minimize risks, and provide patients with transformative results in both function and aesthetics.

## Figures and Tables

**Table 1:** Overview of Thigh Lift (TL) included studies.

Author	N° Patient	Year	Country	Demographic Data	Surgical Technique	Follow Up	Complications	Key Points
The Vertical Medial Thigh Lift **Joseph F. Capella, MD [29]**	335	2014	USA	unspecified	Vertical T-shape TL	12 months	Skin dehiscence Seroma Infection Hematoma Skin necrosis Deep vein thrombosis Pulmonary embolism	Medial thigh lifts that are consistently effective are possible when the vertical and horizontal components of the thigh deformity and their relationship to the lower body are recognized. Our approach to the medial thighs, the correction of lower body deformities before a vertical medial thigh lift with liposuction.
The Concentric Medial Thigh Lift **C. Le Louarn M.D. [9]**	25	2004	France	unspecified	Horizontal medial TL	unspecified	Dehiscence Scar migration Scar widening Insufficient lift effect	The concentric axes of traction, the new incision pattern, the superficial dissection avoiding any dead space and lymphatic damage, the classic anchoring sutures on nonundermined tissue, and the cutaneous excision on demand address our desire for a more reliable and efficient medial thigh lift.
Limb Contouring after Massive Weight Loss: Functional Rather than Aesthetic Improvement **S. Bruschi M.D. [17]**	35	2009	Italy	46 yo	Horizontal/Vertical T-shape TL	12 months	Hematoma Anaemization Seroma Wound dehiscence Haemorrhage Infection Fistula DTV/TEP Abscess	As the follow-up is at least 1 year, we can state that the satisfaction is durable in addition to being high. No reoperation occurred in the period of time occurred and after it to any of the included patients. Nearly all patients declared a relevant esthetic improvement.
Medial Thighplasty: Horizontal and Vertical Procedures after Massive Weight Loss **Labardi L M.D. [10]**	45	2012	Italy	48 yo	15 Horizontal TL; 15 Vertical TL; 15 Vertical T-shape TL	60 months	Scar migration Scar widening	Medial thigh lift surgery is free of major complications, if the basic anatomy of this region is understood, in order to preserve important structures such as the great saphenous vein and femoral vessels. The only complication is the presence of extensive and visible scars along the thigh, in the case of vertical procedure, and along the inguinal canal, with a possible distortion of the labia major, in the case of horizontal procedure.
Liposuction-assisted Medial Thigh Lift in Obese and Non-Obese Patients **Aboueldahab Khalaf Aboueldahab M.D. [11]**	25	2013	Egypt	25–45 yo	20 Horizontal TL 5 T shape TL	12 months	Need for blood transfusion	Liposuction assisted thigh lift improves thigh contour while providing discontinuous thigh undermining. Anchoring of the skin flap to the Colles’ fascia and to the fascia of the adductor muscles reinforces the transverse medial lift and avoids scar descent.
Four-Step Medial Thighplasty **Bryan S. Armijo, M.D. [22]**	45	2014	USA	30 to 67 years	Vertical L shape TL	0.5 months to 9.5 years	Dehiscence Under-correction	The four-step surgical algorithm for medial thighplasty includes: (1) inverted L-shaped anterior markings marked pre-operatively; (2) superwet infiltration of the circumferential thigh; (3) circumferential combined UAL/SAL in an intermediate level except for a key transition to a superficial level in the area to be excised to prevent injury to the saphenous venous system; and (4) pre-designed and patterned skin excision that is rechecked intraoperatively.
An Improved Dual Approach to Post Bariatric Contouring—Staged Liposuction and Modified Medial Thigh Lift: A Case Series **Zaher Jandali M.D. [18]**	21	2014	India	38 yo	modified T-shape TL with buried de-epithelialised dermal flap	6–12 months	Dehischence	The low-seroma rate in this technique is probably due to the reduced of dead space with a buried dermal flap and the combined used to liposuction which generally preserves lymphatic channels.
Medial Thigh Lift in the Massive Weight Loss Population: Outcomes and Complications **Jeffrey A. Gusenoff, M.D. [12]**	106	2015	USA	45.1 ± 10.2 yo	14 Horizontal TL 24 Vertical short T-shape TL 68 Vertical T-shape TL	>70 months	Seroma Dehiscence Bleeding Infection Edema	Complications are highest for the full-length vertical thighplasty (74 percent) and less for the short-scar (67 percent) and horizontal (43 percent) procedures. Age, hypothyroidism, hypertension, and liposuction outside of the area of resection may contribute to postoperative complications.
Personal Evolution in Thighplasty Techniques for Patients Following Massive Weight Loss **Shelly M. Xie M.D. [13]**	65	2017	USA	43–46.1 yo	26 Horizontal TL 39 Vertical T-shape TL	14.6 months	Hematoma Lymphocele/Seroma Erythema Necrosis Dehiscence Infection	Horizontal vector fixation group had a significant increased use of liposuction intraoperatively and a significant decrease in incidence of lymphocele/seroma and infection postoperatively. Our findings reinforce our theorem: limited dissection of the perineum reduces postoperative sequelae while maintaining equivocal wound dehiscence or necrosis.
Risk Factors for Complications after J Medial Thighplasty Following Massive Weight Loss: A Multivariate Analysis of 94 Consecutive Patients **Nicolas Bertheuil, M.D., Ph.D. [23]**	94	2021	France	44 yo	Vertical J-shape TL	12 months	Surgical revision Hospital readmission Emergency room visit Minor complications were those that could be treated in an outpatient clinic Wound dehiscence	In conclusion, we found that a high body mass index and advanced age were independent risk factors for complications of vertical J medial thighplasty.
Concomitant Liposuction Reduces Complications of Vertical Medial Thigh Lift in Massive Weight Loss Patients **Manfred Schmidt, M.D. [19]**	59	2016	Austria	41.5 ± 9.1 yo	Vertical T-shape TL 30 Vertical T-shape TL + liposuction	36 ± 21 months	Seroma Hematoma Wound dehiscence Wound infection Surgical revision	The combined approach (liposuction + vertical medial thighplasty) has led to a significant reduction of complications (seroma, hematoma, surgical revision).
Thighplasty: Improving Aesthetics Through Revival of the Medial, Horizontal Procedure: A Safe and Scar-Saving Option **Karl Schwaiger M.D. [14]**	25	2017	Austria	43 yo	Horizontal TL	32 months	Hematoma Seroma Wound dehiscence Infection Return to OR	Compared to other thighplasty procedures, we observed fewer complications, which indicates a reduction in postoperative morbidity. Therefore, we estimate that the main reason for employing this procedure is the avoidance of the vertical scar and its associated short- and long-term problems and complications (smaller wound surface, better aesthetic appearance, fewer scar irritation, and pain).
Vertical Medial Thigh Lift with the ‘Anchor L Liposculpture’ Technique in Massive Weight Loss Patients: Preliminary results **Özay Özkaya M.D. [24]**	33	2021	Turkey	40.2 ± 10.7 yo	Vertical L-shape TL with dermal flap	21.9 ± 19.4 months	Hematoma Dehiscence Infection Lymphedema Lymphocele Scar migration	Anchor L Liposculpture technique is an easy, apply and reliable technique aimed better surgical results and lower complication rates. Also, it should be considered that an increased amount of liposuction may lead to an increase the complication rates.
L Shaped Lipoplasty **Roberto Bracaglia, MD [25]**	16	2015	Italy	41 yo	Vertical L-shape TL	24 months	Dehiscence Hematoma Lymphedema Lymphocele Wound infection Skin necrosis Tep/TVP	(1) The excess skin and subcutaneous tissue are concentrated exclusively in the anterior medial thigh region (2) The vertical extent is necessary in all patients with moderate to severe laxity and permits a better definition of the medial profile (3) The anchorage to the fascial system of Colles prevents recurrence of ptosis and reduces the surface forces acting on the skin f laps, preventing further risk of dehiscence and necrosis (4) Previous selective liposuction is a crucial preliminary step to prevent vascular and lymphatic complications and to allow only epidermis and dermis resection of the medial thigh area
Medial Thigh Contouring After Massive Weight Loss: The Liposuction-Assisted Medial Thigh Lift (LAMeT) **Verdiana Di Pietro M.D. [20]**	54	2020	Italy	44,16 yo	25 Vertical T-shape TL 26 LAMet	12 months	Seroma Hematoma Wound dehiscence Wound infection Surgical revision Vulva deformation Scar migration	The Liposuction-Assisted Medial Thigh Lift (LAMeT) preserves lymphatic and blood vessels and allows a more anatomical resection of the excess skin. Thus, the incidence of postoperative complications is lower, and the patients heal faster. Complications were observed in 35.7% of the patients that underwent the standard technique and in 3.8% patients that underwent the LAMeT. The most frequent complication was seroma.
Avulsion Thighplasty: Technique Overview and 6-Year Experience **Joseph P. Hunstad, M.D. [15]**	42	2015	USA	49 yo	14 Horizontal TL 28 Vertical T-shape TL	18.3 months	Tep/TVP Hematoma Dehiscence Cellulitis Seroma Lymphedema	Avulsion thighplasty has a low incidence of major complications and minimizes the difficult-to-treat problems of lymphatic origin. It is imperative to counsel patients preoperatively about the high risk of minor complications.
Medial Thigh Lift in Post-bariatric Patients: Our Encouraging Experience **Pierfranco Simone M.D. [30]**	46	2016	Italy	42 yo	vertical T + lipo	12 months	TEP/TVP Wound dehiscence Haematoma Seroma Infection Skin necrosis Lymphoceles Lymphoedema Caudal scar migration Genital distortion Recurrent ptosis	Liposuction with inherent sparing of subcutaneous lymphatics, early mobilisation, and appropriate postoperative management have a pivotal role in reducing complications.
Medial Thigh Contouring in Massive Weight Loss: A Liposuction-Assisted Medial Thigh Lift **Verdiana Di Pietro M.D. [21]**	24	2019	Italy	44.16 yo	Vertical T-shape TL	44 months	Seroma Hematoma Dehiscence Infection Surgical revision	The Liposuction-Assisted Medial Thigh Lift technique preserves the great majority of lymphatics and blood vessels and nerves and allows a more anatomical resection of the excess skin. Thus, it reduces postoperative complications and allows the patient to heal faster.
Fascial Anchoring Technique in Medial Thigh Lift **Lockwood, M.D. [16]**	18	1988	USA	35–45 yo	Horizontal TL	12 months	Infection Hematoma Dehiscence Skin necrosis	Women with very heavy thighs may have an increased risk of recurrence of ptosis. In patients who are marginal candidates for trochanteric liposuction due to decreased skin tone, the medial thigh lift can reduce the upper thigh diameter to an extent that allows a more conservative liposuction of the lateral thigh.

**Table 2:** Overall complication between liposuction and no liposuction. The statistical method used to analyze the differences between the liposuction group and the non-liposuction group was the Chi-square test of independence.

Complication	Liposuction (%)	No Liposuction (%)	*p*-Value
Number of Patients	849	133	n/a
Overall	36.75	70.68	<0.001
TVP	0.12	0.00	1
Re-operation	2.24	3.76	0.45
Hematoma	1.30	6.77	<0.001
Infection	1.77	9.02	<0.001
Scar migration	0.71	7.52	<0.001
Dehiscence	21.20	18.05	0.47
Necrosis	0.24	0.75	0.87
Lymphedema	0.24	0.00	1
Lymphocele/seroma	8.95	24.81	<0.001

## Data Availability

The data supporting the findings of this study are available from the corresponding author upon reasonable request.
